# Lessons from the implementation of a trauma center-based program to support primary care providers in managing opioids and pain after trauma hospitalization

**DOI:** 10.1136/tsaco-2022-001038

**Published:** 2023-02-20

**Authors:** Laura-Mae Baldwin, Laura A Katers, Mark D Sullivan, Debra B Gordon, Adrienne James, David J Tauben, Saman Arbabi

**Affiliations:** 1 Department of Family Medicine and the Harborview Injury Prevention & Research Center, University of Washington, Seattle, Washington, USA; 2 Department of Anesthesiology & Pain Medicine, University of Washington, Seattle, Washington, USA; 3 Department of Psychiatry & Behavioral Sciences, University of Washington, Seattle, Washington, USA; 4 Department of Medicine, University of Washington, Seattle, Washington, USA; 5 Department of Surgery, University of Washington, Seattle, Washington, USA

**Keywords:** implementation, Analgesics, Opioid, Delivery of Health Care, Health Care Quality, Access, And Evaluation

## Abstract

**Background:**

Decreasing exposure to prescription opioids is critical to lowering risk of opioid misuse, overdose and opioid use disorder. This study reports a secondary analysis of a randomized controlled trial implementing an opioid taper support program directed to primary care providers (PCPs) of patients discharged from a level I trauma center to their homes distant from the center, and shares lessons for trauma centers in supporting these patients.

**Methods:**

This longitudinal descriptive mixed-methods study uses quantitative/qualitative data from trial intervention arm patients to examine implementation challenges and outcomes: adoption, acceptability, appropriateness, feasibility, fidelity. In the intervention, a physician assistant (PA) contacted patients after discharge to review their discharge instructions and pain management plan, confirm their PCP’s identity and encourage PCP follow-up. The PA reached out to the PCP to review the discharge instructions and offer ongoing opioid taper and pain management support.

**Results:**

The PA reached 32 of 37 patients randomized to the program. Of these 32, 81% discussed topics not targeted by the intervention (eg, social/financial). The PA identified and reached a PCP’s office for only 51% of patients. Of these, all PCP offices (100% adoption) received one to four consults (mean 1.9) per patient (fidelity). Few consults were with PCPs (22%); most were with medical assistants (56%) or nurses (22%). The PA reported that it was not routinely clear to patients or PCPs who was responsible for post-trauma care and opioid taper, and what the taper instructions were.

**Conclusions:**

This level I trauma center successfully implemented a telephonic opioid taper support program during COVID-19 but adapted the program to allow nurses and medical assistants to receive it. This study demonstrates a critical need to improve care transition from hospitalization to home for patients discharged after trauma.

**Level of evidence:**

Level IV.

**WHAT IS ALREADY KNOWN ON THIS TOPIC**

- Care transitions after trauma hospitalization can be associated with miscommunication or incomplete transfer of information, which has the potential to lead to unintentional persistent opioid prescribing or other deficiencies in post-trauma pain care. Decreasing exposure to prescription opioids is critical to lowering risk of opioid misuse, overdose and opioid use disorder.

**WHAT THIS STUDY ADDS**

- In this study, a level I trauma center successfully implemented an opioid taper support program directed to the primary care providers (PCPs) of patients discharged from a level I trauma center to their homes distant from the center, though only 51% of the discharged patients had a confirmed PCP. Most program consultations were with medical assistants or nurses (78%) rather than PCPs (22%), and these consultations identified that patients and PCPs need a clear taper plan and information on who is responsible for post-trauma care and opioid management after discharge.

**HOW THIS STUDY MIGHT AFFECT RESEARCH, PRACTICE OR POLICY**

- The critical need to improve the care transition from inpatient hospitalization to community for patients discharged after trauma will need a more robust care coordination intervention than that provided in this study, perhaps including both patient-level and provider-level interventions as well as specific payment mechanisms.

## Background

The majority of patients hospitalized with significant trauma receive opioid analgesics at discharge. Although most patients taper off opioids within 6 weeks of hospital discharge,^1 2^ a concerning proportion of patients exposed to opioids for acute pain may experience a serious adverse event or persistent chronic opioid use,^3–5^ a risk factor for development of opioid use disorder (OUD).^1 6^ Additionally, unused quantities of opioids supplied at hospital discharge may be used by others for non-medical use and contribute to opioid-related injuries and deaths.^7^ Patients with trauma may be at particularly high risk for opioid misuse and OUD. One study in two level I trauma centers found that 14% of patients at one center and 61% at the other had at least one risk factor for unintentional opioid overdose and almost half screened positive for substance use.^8^ Decreasing exposure to prescription opioids is an important strategy to decrease risk of opioid misuse, overdose and OUD.

Care transitions after trauma can be associated with miscommunication or incomplete transfer of information,^9 10^ which has the potential to lead to unintentional persistent opioid prescribing or other deficiencies in post-trauma pain care. Standardization of care transitions after trauma hospitalization is currently lacking. Patients with post-trauma pain and opioid use living more distant from their trauma hospital may seek postdischarge care from primary care providers (PCPs) close to their home. However, these PCPs may be unfamiliar with the expected course of recovery after hospitalization for trauma and have less access to resources and expertise to guide management of pain and opioid taper as a patient recovers.

The Harborview Injury Prevention and Research Center conducted a randomized controlled trial of a pilot opioid taper support program between June 2020 and February 2022 to help meet this gap in care. The pilot consisted of a physician assistant (PA) supporting PCPs to manage opioid taper for acute pain after trauma among patients discharged from a level I trauma center to their homes distant from the center. The efficacy results of the randomized controlled trial will be published elsewhere. This study examines the degree to which the trial’s opioid taper support program was implemented as intended and shares key lessons that can be used by trauma centers considering how to best support patients discharged home after a trauma hospitalization.

## Methods

This longitudinal descriptive mixed-methods study uses quantitative and qualitative data gathered by the Collaborative Opioid Taper After Trauma (COTAT) study team to examine implementation challenges as well as outcomes defined as important for assessing intervention implementation: adoption, acceptability, appropriateness, feasibility, and fidelity.

### Collaborative Opioid Taper After Trauma study

The Collaborative Opioid Taper After Trauma (COTAT) study is a clinical trial that randomized patients discharged from a level I trauma center after trauma to usual care or to a program in which their PCPs were supported by a PA pain specialist in managing the patients’ opioid tapers for their pain. Patients recruited to the study were 18 or older, spoke English or Spanish, were admitted to the level I trauma center with an Injury Severity Score of 4 or greater,^11^ and were discharged to their home outside the trauma center’s county. The study intended to recruit equal numbers of patients who were opioid naïve and opioid tolerant (ie, using prescribed opioids in the month before trauma), but the study was unable to do so given challenges that COVID-19 posed on study recruitment. Patients with OUD or evidence of illicit drug use in the past month, active cancer, palliative or hospice care, or serious psychiatric disorders (psychotic symptoms, or a psychiatric hospitalization or suicide attempt in the past year) were excluded. Of the 73 patients providing consent for the trial, 37 were randomized to the PA support intervention for their PCPs; three of these were opioid tolerant.

### Post-trauma hospitalization opioid taper support program

The study intervention or program consisted of an advanced practice provider, namely a PA (LAK), with expertise in pain management to serve as a supportive resource over the phone to a patient’s PCP about post-trauma pain management and opioid tapering for up to 5 months after hospital discharge. The PA had access to a psychiatrist with chronic pain expertise (MS), a family physician (L-MB), and a trauma surgeon (SA) for guidance when providing individual case advice to the PCP. The PA did not directly manage the patient’s care or prescriptions.

The following summarizes the support services planned by the PA:

- Contacting the patient within a few days of hospital discharge to review their discharge instructions and pain management plan, to confirm their PCP’s identity, to assist in identifying a PCP if the patient did not have one, and to encourage follow-up with their PCP.
- Faxing the patient’s discharge summary, discharge instructions, and a detailed study instruction sheet to the PCP within a few days of the patient’s discharge.
- Reaching out to the PCP by phone within the first week after the patient’s discharge to review the discharge instructions, to ensure that the PCP is aware of the support program and its offerings as well as how to reach the PA, and to determine the PCP’s preferences on frequency and preferred method of communication.
- Contacting the PCP’s office weekly for the first 2 weeks after trauma hospitalization discharge, then monthly for up to 5 months to see if the PCP had questions or wanted support in managing their patient’s pain or in tapering their opioid medication. After the first contact, the PA conducted additional consultations only when appropriate (eg, no further consultations were conducted for patients already tapered off opioids or not following up with their PCP).

The PA offered support such as:

- Contacting the hospital trauma team for questions about trauma recovery.
- Advising on the opioid taper plan if not proceeding as planned.
- Problem solving if the PCP had concerns about their patient’s pain management.
- Arranging a case presentation to a telehealth multidisciplinary pain specialist panel about the patient if the PCP desired additional advice.

Early in the support program, the PA discovered that the PCPs often had delegates (eg, medical assistants, nurses) who could receive information and support on their behalf, and these PCP delegates were contacted as alternatives to the PCP, depending on provider availability.

### Data sources

We used five sources of data in this study:

1. Field notes. The PA recorded all interactions with patients, PCPs, and their delegates on forms that were managed using REDCap electronic data capture tools hosted by the Institute of Translational Health Sciences at the University of Washington.^12 13^ Each encounter with the PA was entered on a separate form. These forms included the ability to record answers to both closed-ended and open-ended questions. Forms completed after talking with the patient after discharge included questions related to their follow-up care plan, pain levels, and opioid taper, as well as the opportunity to record other issues that the patient reported in their health since trauma hospitalization. The PA also asked the patient to confirm or identify their PCP. After speaking with the PCP office, the PA recorded: whether the PCP identified themself as the patient’s provider, whether the patient had completed their opioid taper, and whether the PCP needed any support in caring for the patient, particularly related to opioid management.
2. PCP surveys. The study team sent a survey to identified PCPs or their delegates at the end of the intervention, asking six questions concerning the acceptability (eg, ‘I like the COTAT guidance’), appropriateness (eg, ‘The COTAT guidance seems applicable to my practice’), and feasibility (eg, ‘The COTAT guidance seems doable for my practice’) of the intervention (Supplemental Digital Content, online supplemental file 1). These questions were adapted from Weiner *et al*’s Acceptability of Intervention Measure, Intervention Appropriateness Measure, and Feasibility of Intervention Measure.^14^
3. Structured debrief with the PA. The PA was interviewed at the end of the pilot study using semistructured open-ended questions (Supplemental Digital Content, online supplemental file 2). The interview was audiotaped and transcribed, then reviewed by three study team members (L-MB, DBG, MS) to summarize modifications made to the intervention throughout the study, what went well and what the greatest challenges were in implementing the intervention, the responses of patients and providers/delegates to being contacted, the perceived benefits and challenges to the providers about the intervention, and the PA’s perspective on modifying the intervention or on other effective ways to improve opioid management after trauma hospitalization.
4. National Provider Identifier Standard (NPI) data. We used the NPI data to identify provider gender and specialty (family medicine, internal medicine, pediatrics). We used the hospital discharge summary, conversations between the PA and the patient, and online primary care clinic information to identify provider types (Doctor of Medicine [MD], Doctor of Osteopathic Medicine [DO], Advanced Registered Nurse Practitioner [ARNP], PA), practice ZIP code and practice type (eg, federally qualified health center [FQHC], hospital-affiliated clinic). We used rural-urban commuting area codes linked to the primary care clinic ZIP code to categorize practice location into three groups—urban, large rural city/town, and small rural town.
5. Electronic health record data. The electronic health record provided demographic information (ie, age, gender, language preference, residence location, insurance status) on the patients randomized to the intervention, whether or not the patients were admitted to the intensive care unit, hospital length of stay, and information used to calculate the Injury Severity Score.

10.1136/tsaco-2022-001038.supp1 Supplementary data

10.1136/tsaco-2022-001038.supp2 Supplementary data

### Implementation outcomes

We included measures of five implementation outcomes defined by Proctor and colleagues^15 16^ and listed in table 1.

**Table 1:** Implementation outcomes and their definitions, and outcome measures

Implementation outcomes	Definition	Measures
Adoption	Intention, initial decision, or action to try or employ an innovation or evidence-based practice. Adoption may also be called ‘uptake’.	Proportion of participating patients reached by PA interventionist. **Of those whose PCP or PCP office representative was reached and PCP confirmed:** Proportion of PCPs or PCP delegates participating in at least one consult. Type of health professionals participating in the consultations.
Acceptability	Extent to which implementation stakeholders perceive a treatment, service, practice, or innovation to be agreeable, palatable, or satisfactory.	Adaptation of Weiner *et al*’s Acceptability of Intervention Measures (AIM),^14^ measured as agree or strongly agree to the following statements: I like the idea of the Collaborative Opioid Taper After Trauma (COTAT) program. The COTAT program meets my approval.
Appropriateness	Perceived fit, relevance, or compatibility of the innovation or evidence-based practice for a given practice setting, provider, or consumer; and/or perceived fit of the innovation or evidence-based practice to address a particular issue or problem.	Adaptation of Weiner *et al*’s Intervention Appropriateness Measures (IAM),^14^ measured as agree or strongly agree to the following statements: The COTAT program seems applicable to my practice. The COTAT program seems suitable for my practice.
Feasibility	Extent to which a new innovation or practice can be successfully used or carried out within a given agency or setting.	Adaptation of Weiner *et al*’s Feasibility of Intervention Measure (FIM),^14^ measured as agree or strongly agree to the following statements: The COTAT program seems doable for my practice. The COTAT program seems implementable in my practice.
Fidelity	Degree to which an intervention or implementation strategy was delivered as prescribed in the original protocol or as intended by program developers. May include multiple dimensions such as content, process, exposure, and dosage.	**Of those patients with a confirmed PCP and PCP or PCP delegate reached:** Average number and range in number of consultations per provider. Content of discussion at consultation. **Of all patients reached:** Content of discussion between PA and patients. Proportion of patients planning to receive trauma follow-up with different types of providers (ie, trauma center, PCP).

PA, physician assistant; PCP, primary care provider.

The implementation process was measured by identifying barriers, facilitators, and key lessons from the PA’s perspective.

### Analysis

We conducted simple descriptive frequencies of patient and PCP characteristics and the discrete study measures obtained from the PA in the study database. We conducted a content analysis of the open-ended data recorded by the PA, as well as of the PA interview transcription to identify facilitators, challenges, and key learnings related to implementation of the intervention.

## Results

Of the 37 patients randomized to the PA support intervention offered to their PCPs, only 23 (62%) had PCPs. The study team reached and confirmed the identity of the PCP for 19 (83%) of these 23 patients, or 51% of the original cohort of patients randomized to the intervention. One provider cared for two of the patients; thus, our cohorts for examining implementation outcomes of the support program intervention included 18 PCPs caring for 19 patients discharged home distant from the level I trauma center.

### Patient characteristics

The 37 patients had a mean age of 45, were nearly three-quarters male, 95% white, 3% Hispanic, and almost all had an English language preference (table 2). Nearly half were publicly insured, 38% privately insured. They had a mean Injury Severity Score of 13, and almost a quarter spent time in the intensive care unit. The patients’ mean hospital length of stay was 4.5 days. Just over 40% of the patients lived in rural areas.

**Table 2:** Characteristics of patients randomized to the support intervention

Characteristics	Patients randomized to the intervention n=37
Age, mean (SD)	45.4 (17.3)
Gender (% male)	73
Race (%)	
White	95
Black/African American	5
% Hispanic	3
% with English language preference	97
Injury Severity Score, mean (SD)	13.0 (8.5)
% with intensive care unit admission	22
Hospital length of stay, mean (SD)	4.5 (3.4)
Residence location* (%)	
Urban	59
Large rural city/town	27
Small rural town	11
Isolated small rural town	3
Primary insurance (%)	
Private insurer	38
Workers’ compensation (Labor and Industries)	8
Medicaid	22
Medicare	24
Other†	8

Some percentages do not add up to a total of 100% due to rounding error.

*Residence location is defined using rural-urban commuting area codes linked to the ZIP code of the patient’s residence.^34 35^

†Other included Tricare, Indian Health Service, unknown.

### PCP characteristics

The 18 PCPs were largely family physicians, and nearly half were female (table 3). Nearly a quarter of the PCPs were advanced practice providers (three ARNPs, one PA) with the remainder MDs and DOs. Almost 40% of the PCPs’ practice locations were in rural locations. The largest proportion of PCPs practiced in FQHCs (33%), followed by independent practices and hospital/health system-affiliated clinics. The minority practiced in integrated health systems.

**Table 3:** Characteristics of confirmed* primary care providers (PCPs) with intervention patients hospitalized with trauma distant from their homes

Characteristics	Primary care providers n=18
Specialty (%)	
Family medicine	78
Internal medicine	17
Pediatrics	6
Gender (% male)	56
Provider type (%)	
MD/DO	78
ARNP	17
PA	6
Geographic location† (%)	
Urban	61
Large rural city/town	22
Small rural town	17
Practice type (%)	
FQHC	33
Independent practice	28
Hospital/health system affiliated	28
Integrated health system	11

Some percentages do not add up to a total of 100% due to rounding error.

*Confirmed PCPs are those for whom the PCP or the PCP’s office representative was reached and the study team confirmed that this was the patient’s PCP.

†Geographic location was categorized using the rural-urban commuting area (RUCA) codes of PCP’s work ZIP code.

ARNP, Advanced Registered Nurse Practitioner; DO, Doctor of Osteopathic Medicine; FQHC, federally qualified health center; MD, Doctor of Medicine; PA, physician assistant.

### Adoption of and fidelity to the support program by the patients

The PA reached 32 (86%) of the 37 participating patients by phone after discharge from the trauma center (adoption), and when reached, the PA documented that they consistently discussed the planned content for the call (pain management, opioid and other medication management, trauma hospitalization follow-up, and making an appointment with a PCP; measure of fidelity). The PA documented that the vast majority of patients (26 of the 32 patients, 81%) discussed topics beyond the planned content, the most common being social context or financial issues (72%), more pain than expected (47%), opioid side effects (28%), aftercare with other providers (25%), and issues with mood or well-being (25%). Half of the patients were not planning to see a PCP, citing various reasons: not wanting to travel to the PCP office, competing priorities (eg, family member with more pressing medical concerns), feeling that follow-up at the trauma center was adequate and logistical difficulties (eg, insurance, time off work).

### Adoption of and fidelity to the support program by the PCPs

The PA support program was provided to all of the PCPs caring for the 19 patients whose PCP was reached and confirmed (100% adoption, table 4). The PA completed at least one consultation with a PCP or PCP delegate on behalf of each of these patients (fidelity). The number of consultations ranged from one to four, with an average of 1.9 (fidelity). A total of 36 consultations were conducted over the course of the study. The minority of consultations were conducted with PCPs (22%); most were conducted with PCP delegates (56% with medical assistants, 22% with nurses). The PA documented that the three topics planned for the consultations (pain management, opioid management, and trauma hospitalization follow-up) were discussed for the majority of the patients (fidelity). Other topics that the PA documented as discussed during the consultations with PCPs or their delegates included other medication management, aftercare with other providers, opioid side effects, and social context or financial issues.

**Table 4:** Implementation outcomes of the support program: consultations with PCPs

Measure	Implementation outcome measured	n	%
PCP or PCP office representative reached and PCP confirmed		19	
Proportion whose PCP/PCP delegate participated in at least one consultation with the PA	Adoption	19	100
Average number (range) of consultations on behalf of each patient	Fidelity	1.9 (1–4)	
Total number of consultations		36	
**Of the 36 consults with a PCP or PCP delegate**			
Types of healthcare professionals participating in the consultations	Fidelity		
Medical assistant		20	56
PCP (MD, DO, PA, ARNP)		8	22
Nurse		8	22
Documented content of discussion during consults	Fidelity	n=19	
*Planned consult content*			
Pain management		13	68
Opioid management		15	79
Trauma hospitalization follow-up management		15	79
*Unplanned consult content*			
Other medication management		4	21
Aftercare with other providers		3	16
Opioid side effects		2	11
Social/financial context or issues		2	11
Mood issues or adverse well-being		1	5

ARNP, Advanced Registered Nurse Practitioner; DO, Doctor of Osteopathic Medicine; MD, Doctor of Medicine; PA, physician assistant; PCP, primary care provider.

### Acceptability, appropriateness, and feasibility of the support program

The survey of PCPs or their delegates conducted after completion of the support program (data not shown) had a low response rate (18%, 3 of the 17 surveyed). Two of the three responding PCPs agreed that the program was acceptable, appropriate, and feasible, and the third was neutral about the program.

### Facilitators and barriers to implementing the support program

The PA’s work in the support program was facilitated by a high level of receptivity by the patients, and by the shift to providing the support to the PCPs directly, as they were hard to reach, and to the PCP delegates (table 5). The PA cited many challenges to providing support. First, the study took place exclusively during the COVID-19 pandemic, which decreased the ability to meet patients in the hospital prior to discharge, the willingness of patients to see their PCPs due to COVID-19 concerns, and the availability of the PCPs. Second, PCPs were difficult to reach, and the PA was not available at all times for call backs from the providers. Third, many patients did not plan to follow-up with their PCP, limiting the utility of a PCP-focused intervention. Finally, the PA expressed concern that the PA support could be construed as the trauma center trying to provide oversight of the PCP’s patient care, and that it was challenging to recommend a care plan that differed from that already offered by the PCP.

**Table 5:** Facilitators and challenges the PA interventionist reported in implementing the support program

Facilitators	
**Patient level**	
Patient receptivity	Patients were receptive and appreciative of post-trauma hospitalization follow-up.
**Provider level**	
Use of provider delegates	Ability to deliver intervention to PCP delegates provided the opportunity to reach PCPs indirectly.
Provider/delegate receptivity	Providers and their delegates were generally receptive to the intervention.

Challenges	
**Patient level**	
COVID-19	PA was not able to develop rapport through a first in-person visit with the patient. Patients were less likely to follow-up with their PCP due to COVID-19 concerns (eg, telehealth only, limited hours).
PA availability	PA was not available 24/7, so often did not reach patient prior to discharge. Reaching patients after discharge could be challenging.
Many patients did not plan to follow-up with PCP.	The intervention was with the PCP, so if the patient did not plan to see their PCP, there would be no opportunity to benefit from the intervention. Patients had various reasons they did not plan to follow-up with the PCP (eg, planning follow-up at trauma center, lack of understanding of PCP role in post-trauma hospitalization care, competing priorities at home such as child with OUD or spouse entering hospice).
**Provider level**	
COVID-19	Providers were less available for the intervention due to factors such as the busyness of COVID-19, abbreviated hours, reduced staff.
PA availability	The PA interventionist was not always available and could miss provider call back for consultation.
Providers hard to reach	There was often no provider back line, or the provider had left the practice.
PA concerns	The PA found it challenging to discuss a different care plan from the one that the provider had already offered the patient (eg, opioid prescribing from more than one provider). The PA was concerned that the providers might think the trauma hospital was trying to provide oversight of patient care.

OUD, opioid use disorder; PA, physician assistant; PCP, primary care provider.

### Key lessons for trauma centers

Several key lessons emanated from the interview with the PA (table 6). First, patients were easier to reach than PCPs, and were appreciative of post-trauma hospitalization follow-up. They discussed many post-trauma issues beyond pain and opioid management. Patients discharged home after trauma often did well after hospitalization, tapered off their prescribed opioids early, and their providers may not have needed a post-trauma hospitalization intervention. Notably, it was not routinely clear to patients or PCPs who was responsible for post-trauma care and the opioid taper, and taper instructions were also not clear to many patients. Finally, patients were receptive to and routinely used non-opioid options for pain management after trauma.

**Table 6:** Key lessons in implementing a support program for patients with trauma after discharge

Key lessons	Implications for a post-trauma hospitalization support program
Many patients do well after trauma discharge and their primary care providers may not need a post-trauma hospitalization intervention.	Identify higher risk patients for a post-trauma hospitalization discharge intervention.
It is not always clear to patients or to the primary care providers who is responsible for post-trauma care and opioid prescribing/taper.	Include clear contact information for who is responsible for opioid management and post-trauma hospitalization care on the discharge summary.
Opioid tapering instructions are not clear to many patients.	Provide clearer post-trauma hospitalization tapering instructions as part of discharge, including on the discharge summary.
Patients were easier to reach than primary care providers and were very receptive to contact with the PA. Patients appreciated additional support after a trauma hospitalization and raised issues beyond pain and opioid management.	Test a multilevel intervention directed at both patients and primary care providers.
Patients were actively using and receptive to non-opioid options for pain control after trauma hospitalization discharge.	Efforts to limit access to post-trauma opioids need to be matched by efforts to provide access to non-opioid options for pain control.

PA, physician assistant.

## Discussion

All PCP offices that the PA contacted accepted this study’s supports as they tapered opioid medications among patients discharged home from a level I trauma center. It was challenging, however, for the program to have its intended reach. Half of the study patients with trauma did not have identifiable PCPs, and of those patients who did, many did not plan to follow-up with their PCPs. The program demonstrated fidelity to the intervention by delivering its core elements of support for pain and opioid management and post-trauma hospitalization follow-up, but it was not feasible to deliver the support to the intended PCP recipients. Instead, the intervention was largely delivered to a PCP delegate on their clinical team, such as a medical assistant or nurse, potentially attenuating the strength of the intervention.

Because this intervention’s goal was to support prompt, appropriate taper of opioid medications, it was directed toward the PCP opioid prescribers. Several factors made delivery of the intervention directly to PCPs difficult. First, the intervention was conducted during the COVID-19 pandemic, and primary care practice was seriously disrupted.^17–20^ Many PCP offices decreased hours and either cut back on or lost staff support during this time.^21 22^ Second, primary care practice has increasingly become a team-based system of care^23 24^; thus, it is not surprising that the PCPs’ delegates on their clinical teams often received the intervention. Third, a number of patients either were promptly tapering off opioids themselves or chose not to follow-up with their PCPs. The PCPs’ clinical staff members were able to share this information with the PA and gather information from the PA on how to reach the support program if needed, making direct contact with the PCP less important. Future interventions would benefit from targeting only those patients at highest risk for difficulty in tapering opioids, as well as from tailoring the intervention content based on whether the recipients are the opioid prescribers themselves, or delegates from their team.

One of the most notable study findings was that only half of the patients discharged to their homes after trauma had a confirmed PCP. This may relate to the patients being largely younger and male, both factors known to decrease the likelihood of having a PCP.^25^ Further, among patients with confirmed PCPs, the consulting PA found that both PCPs and patients were uncertain about who was responsible for postdischarge care and opioid prescribing. This highlights the void that many patients with trauma experience when discharged from the level I trauma center. For those with a PCP, patients reported lack of clarity on the timing of the transition back to their PCP’s care, and which aspects of the patient’s care should be handled by the PCP. Patients were receptive and appreciative of the PA contact call after discharge, and the vast majority (81%) discussed topics beyond the planned content of the one contact call with the PA. The most common topics were social context, financial issues, having more pain than expected, opioid side effects, and issues with mood or well-being. These findings contribute to a scarce literature about the experience of patients with trauma in the inpatient to outpatient care transition. A recent study interviewing 13 patients with orthopedic trauma identified the theme of insecurity after discharge due to unmet information needs about their injury and its expected effect on their physical function, about the psychological reaction to trauma, and about opioid side effects and tapering.^26^ These patients also noted lack of follow-up after discharge from the trauma center as a concern. Zatzick *et al* tested two models of care seeking to improve the postdischarge experience of patients with trauma—identification of patient concerns prior to discharge followed by either a care management program or nurse notification of the concerns.^27^ This study identified high rates of concern related to physical health, work and finances, psychological health, and social well-being (eg, of family and friends) both before and after discharge. Our study’s results are consistent with the patient concerns identified in these studies.

### Implications for level I trauma centers

Hospital discharge services routinely include development of a discharge summary that is sent to the patients’ follow-up care providers.^28^ Ensuring that these discharge summaries clearly and specifically designate the provider responsible for post-trauma care and opioid management after discharge and a clear opioid taper plan would be a first step toward supporting patients and their PCPs. Discharge care coordinators who facilitate the discharge planning for inpatients could take responsibility for ensuring that this tailored information is provided consistently in all discharge summaries. Second, Zatzick *et al*’s care management program for patients both prior to and after trauma discharge resulted in fewer serious concerns in the 6 months after trauma discharge as well as fewer emergency department visits 3–6 months after discharge.^27^ Trauma hospitalizations are frequently complex and require multiple services (eg, multiple surgeons, pain management specialists, physical and occupational therapists) to optimize outcomes. This complex care from multiple services can lead to complex discharge management needs, making a navigator like a care coordinator critical for patients after discharge. For post-trauma patients who live distant from the trauma center and are at high risk of adverse outcomes, this can be even more important. Testing a multilevel intervention that includes both a patient-level intervention with a navigator or care manager and a PCP-level intervention with an opioid management component would shed light on whether Zatzick’s care management intervention findings could be replicated and perhaps amplified for this selected group. Preliminary investigation supports the feasibility of a patient-level care management intervention for patients with OUD.^29^

### Implications for policy makers seeking to optimize post-trauma patient care

The American College of Surgeons Committee on Trauma publishes clinical best practice guidelines that inform operation of US trauma centers and link trauma center designation to quality indicators.^30^ The American College of Surgeons’ Resources for Optimal Care of the Injured Patient (2022 Standards)^31^ includes a section on discharge planning, which requires that all trauma centers have a process to determine the level of care and the rehabilitation services required after trauma center discharge. These standards recommend, but do not require, that level I and II trauma centers adopt patient-centered strategies for facilitating patient transition into the community. They include ongoing care management as one of those strategies, citing Zatzick *et al*’s research.^27^ Shifting this recommendation to a requirement that includes care coordination, especially for high-risk patients prescribed opioids after discharge, and then monitoring the requirement’s implementation and outcomes would help ensure that level I trauma centers include a post-trauma discharge program for supporting their patients as they transition from inpatient to their home settings. Additionally, high-quality post-trauma discharge care coordination requires funding. Current payment structures for global surgical care, including the surgical hospitalization and postoperative outpatient follow-up care, do not support the cost of this type of service. Development of new billable codes or value-based allocation of existing bundled payments for perioperative care management services to support high-risk populations would be critical to implementing a comprehensive inpatient and care transition program.^32^

### Limitations

This study is limited by the relatively small number of trauma patients recruited to the support program’s intervention. Recruitment was conducted during the COVID-19 pandemic, which impacted recruitment as well as the primary care practice landscape throughout the study. Despite this disruption, the study had 100% adoption of the intervention by the PCPs or their delegates who were confirmed and reached. The trial initially had planned to recruit a stratified sample of patients who were opioid naïve versus patients using opioids chronically for pain. However, few patients using opioids chronically for pain qualified for the study, so we were unable to examine the implementation of the support program in this higher risk population. Although 2 of the 3 PCPs responding to a postintervention study survey thought that the support program was acceptable, appropriate, and feasible, the survey response rate was low—18%. Finally, implementation of the program was complicated by a new set of state rules concerning opioid prescribing that went into effect around the same time as the study. These rules refer to guidelines that in opioid-naïve patients, any opioids prescribed during the first 6 weeks postoperatively should be managed solely by the surgeon.^33^ This may have led some patients to contact one of several surgical services involved in their inpatient care for pain management issues rather than their PCPs, even if they lived more distant from the trauma center, although we did not measure this.

## Conclusions

This pilot program was successfully implemented during the early stages of the COVID-19 pandemic to support PCPs in tapering opioid medications prescribed to patients with trauma who were discharged to their homes distant from the level I trauma center. The program was hampered by the fact that nearly half of the discharged patients did not have a confirmed PCP, and future interventions will need to take this into account. For those PCPs who could be identified and confirmed, adoption and fidelity to the program’s planned intervention were nearly complete. Implementing the intervention with PCPs themselves was not always feasible, however, requiring an adaptation to the intervention—allowing the intervention recipient to be a clinical team member as an alternative to the PCP. Beyond this study’s implementation outcomes, it has illuminated a critical need to improve the care transition from inpatient hospitalization to community for patients discharged after trauma, both to attend to opioid management and to many other concerns that patients with trauma experience.

## Data Availability

Data are available upon reasonable request.
