# Supplementary material for: Lessons from the implementation of a trauma center-based program to support primary care providers in managing opioids and pain after trauma hospitalization
Source: Trauma Surg Acute Care Open. 2023 Feb 20;8(1):e001038. doi: 10.1136/tsaco-2022-001038 (PMC9944266; doi:10.1136/tsaco-2022-001038)
Supplement: Supplementary data [file tsaco-2022-001038supp001.pdf]

Approved
12/4/2020
UW IRB

Supplemental Digital Content
Supplementary File 1: Post-Intervention Survey of identified PCPs or their delegates about the acceptability, appropriateness, and feasibility of the COTAT intervention

Please answer the 6 questions below about the appropriateness, feasibility, and acceptability of the Collaborative Opioid Taper After Trauma (COTAT) study guidance to you and your practice.

1] The COTAT guidance seems applicable to my practice:

1	2	3	4	5
Completely Disagree	Disagree	Neither agree nor disagree	Agree	Completely Agree

2] The COTAT guidance seems doable for my practice:

1	2	3	4	5
Completely Disagree	Disagree	Neither agree nor disagree	Agree	Completely Agree

3] I like the COTAT guidance:

1	2	3	4	5
Completely Disagree	Disagree	Neither agree nor disagree	Agree	Completely Agree

4] The COTAT guidance seems implementable in my practice:

1	2	3	4	5
Completely Disagree	Disagree	Neither agree nor disagree	Agree	Completely Agree

5] The COTAT guidance meets my approval:

1	2	3	4	5
Completely Disagree	Disagree	Neither agree nor disagree	Agree	Completely Agree

6] The COTAT guidance seems suitable for my practice:

1	2	3	4	5
Completely Disagree	Disagree	Neither agree nor disagree	Agree	Completely Agree
