# Supplementary material for: Lessons from the implementation of a trauma center-based program to support primary care providers in managing opioids and pain after trauma hospitalization
Source: Trauma Surg Acute Care Open. 2023 Feb 20;8(1):e001038. doi: 10.1136/tsaco-2022-001038 (PMC9944266; doi:10.1136/tsaco-2022-001038)
Supplement: Supplementary data [file tsaco-2022-001038supp002.pdf]

Supplemental Digital Content

Supplementary File 2: Structured debrief guide with interview questions asked of the PA interventionist after completion of the COTAT intervention

Thank you for meeting with me to discuss your experience implementing the intervention for the Collaborative Opioid Taper After Trauma study. We will ask 20 open-ended questions about your experience. When you answer the questions I'll pose, please focus on your experiences in conducting the intervention that included contacting patients after trauma hospitalization and supporting their primary care providers in managing their patients' opioids post-hospitalization. This interview is not about your experiences in completing the research aspects of this work (e.g., completing REDCap forms, participating on the study team).

1. Please describe the intervention as you understood it at the beginning of the study.

2. Please describe any modifications that were made to the intervention throughout the study, either because the research team made a change, or because you were unable to accomplish the intervention as intended.
    a. For each modification, note any story about how it was made

3. What went well as you implemented the intervention?
    a. In contacting patients
    b. In contacting providers
    c. In the consultation process

4. What were the greatest challenges you experienced in implementing the intervention?
    a. In contacting patients
    b. In contacting providers
    c. In the consultation process

5. What would have made implementing the intervention easier for you?
    a. In contacting patients
    b. In contacting providers
    c. In the consultation process

6. From your perspective and experiences, how did patients respond to your contacting them?

7. A. What kinds of benefits did patients mention to you related to the intervention?

B. From your perspective and experience, are there other benefits, either intentional or unintentional, that you feel patients experienced related to the intervention?

8. A. What types of patients were more likely to either mention benefits to you or that you noticed benefitted more from the intervention?

   B. What types of patients were either less likely to mention benefits to you or that you noticed benefitted less from the intervention?

9. A. What kinds of challenges did patients mention to you related to the intervention?

   B. From your perspective and experience, are there other challenges, either intentional or unintentional, that you feel patients experienced related to the intervention?

10. A. What types of patients were either more likely to mention challenges to you or from your perspective were more likely to experience challenges related to the intervention?

    B. What types of patients were either less likely to mention challenges to you or from your perspective were less likely to experience challenges related to the intervention?

11. From your perspective and experiences, how did providers respond to your contacting them? How did they respond to the intervention when you were able to deliver it? What do you think providers might say about this intervention? (Prompt for both benefits and challenges)

12. How about provider delegates? How did the delegates respond to your contacting them? How did they respond to the intervention when you were able to deliver it? What do you think provider delegates might say about this intervention? (Prompt for both benefits and challenges)

13. A. What kinds of benefits did providers or provider delegates mention to you related to the intervention?
    NOTE: Please distinguish whether providers or provider delegates as Katers answers the question

B. From your perspective and experience, are there other benefits, either intentional or unintentional, that you feel providers or provider delegates experienced related to the intervention?
NOTE: Please distinguish whether providers or provider delegates as Katers answers the question

14. A. What types of providers or provider delegates were more likely to either mention benefits to you or that you noticed benefitted more from the intervention?
NOTE: Please distinguish whether providers or provider delegates as Katers answers the question

    B. What types of providers or provider delegates were either less likely to mention benefits to you or that you noticed benefitted less from the intervention?
NOTE: Please distinguish whether providers or provider delegates as Katers answers the question

15. A. What kinds of challenges did providers or provider delegates mention to you related to the intervention?
NOTE: Please distinguish whether providers or provider delegates as Katers answers the question

    B. From your perspective and experience, are there other challenges, either intentional or unintentional, that you feel providers or provider delegates experienced related to the intervention?
NOTE: Please distinguish whether providers or provider delegates as Katers answers the question

16. A. What types of providers or provider delegates were either more likely to mention challenges to you or from your perspective were more likely to experience challenges related to the intervention?
NOTE: Please distinguish whether providers or provider delegates as Katers answers the question

    B. What types of providers or provider delegates were either less likely to mention challenges to you or from your perspective were less likely to experience challenges related to the intervention?
NOTE: Please distinguish whether providers or provider delegates as Katers answers the question

17. You weren't able to accomplish the intervention as intended with many of the providers. What do you think are the reasons for this? Which of these reasons are the most important ones?

18. If you could modify the intervention knowing what you know now, how would you change it?

19. Given your experience with this intervention, what do you think might be the most effective ways to improve opioid management after discharge from a trauma hospitalization?

    Prompt with this question after the open-ended question above: Do you think decreasing opioid supply (decreased prescribing at discharge) or decreasing opioid demand (increasing access to opioid alternatives) will be most important?

20. What else would you like to share about this intervention from your perspective and experience?
